# Peculiar Presentation of Gastrointestinal Stromal Tumor in a Patient With Early Satiety

**DOI:** 10.7759/cureus.36523

**Published:** 2023-03-22

**Authors:** Franklin Obi, Ricardo Anguiano-Albarran, Daniel Cain, Steven Mudrovich, Melvin Simien

**Affiliations:** 1 Internal Medicine, Baylor Scott & White All Saints Medical Center, Fort Worth, USA; 2 Medicine, Baylor Scott & White All Saints Medical Center, Fort Worth, USA; 3 Interventional Endoscopy, Baylor Scott & White Digestive Diseases, Fort Worth, USA

**Keywords:** adult gastroenterology, eus, gastrointestinal stromal tumor, pancreatic mass, gist

## Abstract

Gastrointestinal stromal tumors (GISTs) are one of the most common, potentially malignant, subepithelial lesions identified in the gastrointestinal tract. Hypothesized to derive from the interstitial cells of Cajal (ICC), GISTs commonly demonstrate gain of function mutations in proto-oncogenic receptor tyrosine kinase CD117 (KIT). Depending on mitotic activity and tumor size characteristics, GISTs may transform from benign to malignant neoplasms. Increasing evidence suggests that early identification of a GIST is paramount for optimal prognostic outcomes. We present a rare case of a GIST located in the uncinate pancreas identified via endoscopic ultrasound (EUS) and diagnosed with an EUS-guided fine needle aspiration (EUS-FNA) biopsy.

## Introduction

Pancreatic lesions can be distinguished with respect to their morphologic features via imaging. The differential of pancreatic lesions is broad and includes solid adenocarcinoma tumors, mucinous cystic neoplasms, mixed cystic and solid lesions, intraductal papillary mucinous neoplasms, microcystic lesions, and, very rarely, stromal tumors. Spindle cell stromal tumors in the gastrointestinal tract are identified as gastrointestinal stromal tumors (GISTs). Most commonly, these lesions are found in the distal stomach and proximal small bowel. GISTs arising outside of the traditional bowel lumen are termed extra-gastrointestinal stromal tumors (eGIST). It is estimated that the incidence of GISTs is between 7 to 14 cases per million per year [1-4]. Even more sparse is the incidence of eGISTs. To date, less than 40 cases have been reported in the literature [5].

These tumors are believed to derive from the interstitial cells of Cajal (ICC) due to KIT protein positivity in this cell line [2,6]. Up to 80% of GISTs have an activating mutation in KIT [2,7]. KIT-negative GISTs have been identified, and up to 5-8% of those demonstrate platelet-derived growth factor receptor-α (PDGFR-α) mutations [7,8]. With predisposing oncogenic mutations, it is reported that 10-30% of GISTs have a malignant course [1,9,10]. Overall prognosis is dependent on the mitotic index, tumor size, and presence of metastatic disease at the time of diagnosis [1,11]. Mietinen et al. reported increased metastatic risk correlating with tumor size [10]. These tumors should be stratified based on the risk of malignancy. It is recommended to not strictly classify lesions as benign or malignant. Diagnosis is confirmed via immunohistochemical analysis for KIT-CD117, PDGFR-α, and/or CD34 staining positivity [12,13]. Due to the complex variables contributing to malignant potential and extensive diagnostic immunohistochemical testing, early diagnosis of GISTs is paramount.

As opposed to common gastrointestinal tract mucosal pathology, traditional endoscopic forceps biopsy has a limited role in accurately diagnosing GIST lesions. GISTs are subepithelial in origin, and mucosal sampling can make immunohistochemical analysis difficult. A multimodality approach is often necessary for patients when there is clinical suspicion of malignant pancreatic tumors. Endoscopic ultrasound-guided fine needle aspiration (EUS-FNA) and magnetic resonance imaging (MRI) can provide further detailed information for stratification. Endoscopic ultrasound (EUS) and EUS-FNA are critical components of evaluation and, ultimately, diagnosis. EUS-FNA has been demonstrated to be the most accurate, safe, and reliable method to secure a definitive diagnosis [1,3,14,15].

## Case presentation

A 69-year-old female presented with vague abdominal pain, early satiety, decreased appetite, and concomitant 10lb weight loss over the course of two months. Other than controlled hypertension and stable depression, the patient did not have further comorbidities. About two weeks prior to referral to our facility, the patient was seen at an outside hospital emergency department for intractable nausea, vomiting, and diarrhea. She was subsequently diagnosed with gastroenteritis and sent home. When she presented to our clinic, she had mild tenderness in the right upper quadrant and epigastric area upon palpation, with no distention, scleral icterus, jaundice, or decrease in bowel sounds. Initial labs were unremarkable, but weight loss and ongoing symptoms after gastritis treatment had been initiated prompted further evaluation with imaging.

**Figure 1:**
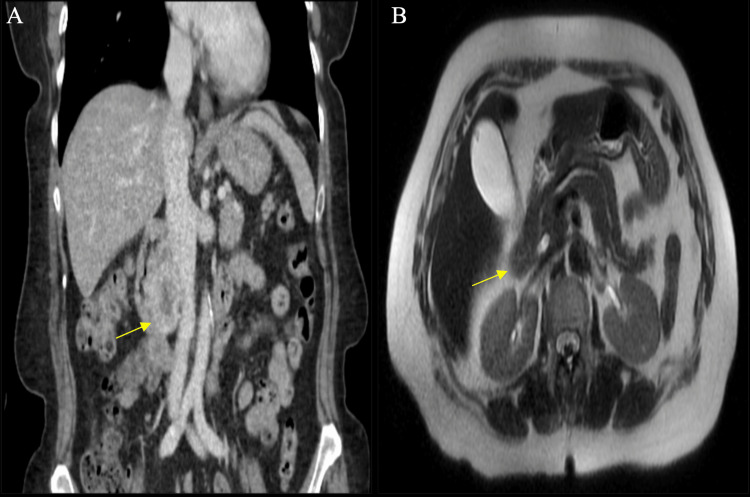
Initial CT and MRI abdominal imaging demonstrating a pancreatic mass A: CT sagittal view of mass. B: MRI demonstrating heterogenous enhancing mass within proximity of right renal vasculature and adjacent duodenum

Initial evaluation of the patient with repeat computed tomography (CT) of the abdomen identified a mass in the pancreatic head (Figure 1A). This finding was followed up with magnetic resonance cholangiopancreatography (MRCP) and MRI with and without contrast of the abdomen. MRCP demonstrated no abnormal ductal dilatation or strictures, a normal gallbladder, liver, and spleen. MRI-abdomen demonstrated a heterogenous enhancing mass measuring approximately 4cm x 2.9cm in the pancreatic head with abutment of the adjacent duodenum and inferior vena cava (Figure 1B).

**Figure 2:**
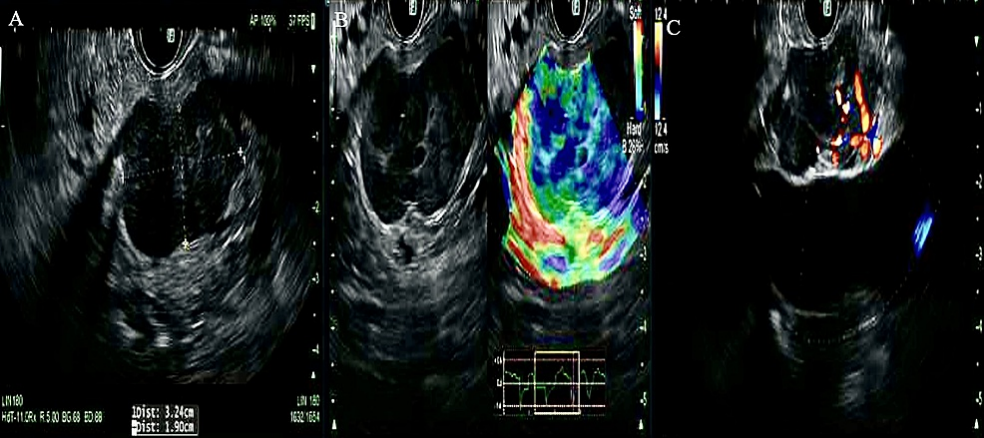
Endosonographic findings A: Hypoechoic mass in the uncinate process with measurements in maximal cross-sectional diameter noted. B: Elastography view of mass prior to fine needle aspiration. C: Intact interface between mass and celiac trunk suggesting lack of vascular invasion

The decision was then made to proceed with EUS-FNA, given the degree of clinical suspicion for malignancy. EUS found a hypoechoic oval mass in the uncinate process of the pancreas without evidence of vascular invasion (Figure 2).

**Figure 3:**
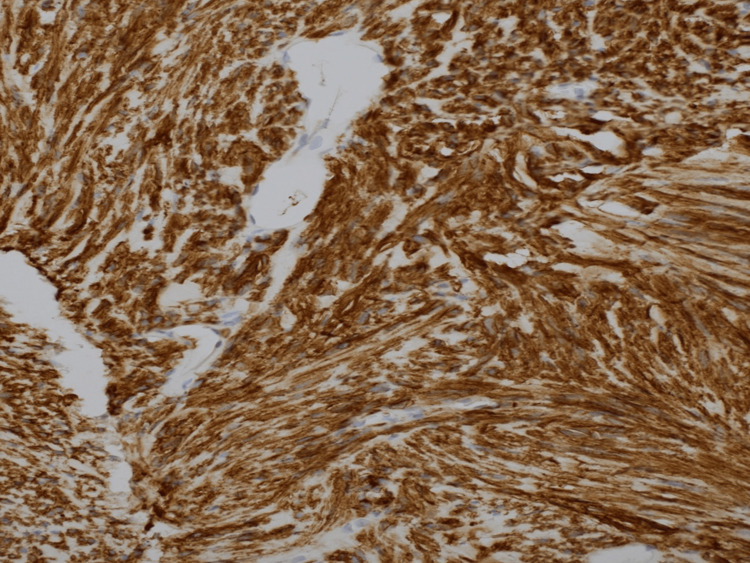
Core tissue biopsy demonstrating uniform positivity for CD117

Subsequent core needle biopsy results identified gastrointestinal stromal tumor pathology. Initial histopathologic examination demonstrated strong positive staining for CD117 (Figure 3). KIT mutation analysis was positive for a DNA sequence change on exon 11. Studies have suggested that tumors with this mutation pattern are responsive to tyrosine kinase inhibitor therapies [16-19].

## Discussion

The present standard of care, based on the limited number of reported cases, is for evaluation for surgical resection with neoadjuvant or adjuvant chemotherapy [5]. Fletcher et al. proposed a risk of aggressive behavior scoring system for GIST. The parameters of the scoring system were size (ranging from <2cm to >10cm) and mitotic count (ranging from <5/50 high power field (HPF) to >10/50 HPF) [20]. This risk stratification score, as well as tumor location and mutations, can then be used to determine the best course for therapy. Imatinib, a tyrosine kinase inhibitor active against KIT-CD117 mutations that are prevalent in GISTs, is the leading medical therapy [5,9,16,19,20]. It has shown benefits with use as either neoadjuvant and adjuvant chemotherapy and can decrease tumor burden prior to resection [21]. The use of imatinib has a median five-year survival in GIST cases [22]. The most definitive treatment and goal for GISTs and eGISTs is surgery. Required surgery type is largely based on the location of the tumor, with clear microscopic margins being the desired outcome [5].

The patient in this case was initiated on imatinib. A subsequent positron emission tomography (PET) scan has found no evidence of metastasis. Serial imaging, while on systemic therapy, has demonstrated positive tumor shrinkage. Ultimately, the patient is being evaluated for imatinib response with plans for duodenal sleeve resection versus the Whipple procedure. This case demonstrates the utility of EUS-FNA in diagnosing gastrointestinal pathology. The primary aspect gleaned from this case is that early diagnosis without vascular invasion and metastasis provides the patient with the best possible outcomes.

## Conclusions

In conclusion, this case is a valuable addition to the literature for further diagnostic approaches in working up extra-gastrointestinal stromal tumors. The benefit of using EUS-FNA is that it allows for accurate sampling of these tumors even when in areas that are difficult to access. Rapid identification and sampling allow for timely evaluation of medical and/or surgical therapy, and thus, better patient outcomes.
